# Identification of Differentially Expressed Long Non-coding RNAs in Polarized Macrophages

**DOI:** 10.1038/srep19705

**Published:** 2016-01-22

**Authors:** Zikun Huang, Qing Luo, Fangyi Yao, Cheng Qing, Jianqing Ye, Yating Deng, Junming Li

**Affiliations:** 1 Department of Clinical Laboratory, the First Affiliated Hospital of Nanchang University, Nanchang 330006, Jiangxi, China; 2 Department of Clinical Laboratory, Jiangxi Province Blood Center, Nanchang 330006, Jiangxi, China; 3 Intensive Care Unit, the First Affiliated Hospital of Nanchang University, Nanchang 330006, Jiangxi, China

## Abstract

Macrophages display remarkable plasticity, with the ability to undergo dynamic transition between classically and alternatively activated phenotypes. Long non-coding RNAs (lncRNAs) are more than 200 nucleotides in length and play roles in various biological pathways. However, the role of lncRNAs in regulating macrophage polarization has yet to be explored. In this study, lncRNAs expression profiles were determined in human monocyte-derived macrophages (MDMs) incubated in conditions causing activation toward M(IFN-γ + LPS) or M(IL-4) phenotypes. Compared with primary MDMs, 9343 lncRNAs and 5903 mRNAs were deregulated in M(IFN-γ + LPS) group (fold change ≥2.0, *P* < 0.05), 4592 lncRNAs and 3122 mRNAs were deregulated in M(IL-4) group. RT-qPCR results were generally consistent with the microarray data. Furthermore, we found that TCONS_00019715 is expressed at a higher level in M(IFN-γ + LPS) macrophages than in M(IL-4) macrophages. TCONS_00019715 expression was decreased when M(IFN-γ + LPS) converted to M(IL-4) whereas increased when M(IL-4) converted to M(IFN-γ + LPS). Knockdown of TCONS_00019715 following the activation of THP-1 cellls using IFN-γ and LPS diminished the expression of M(IFN-γ + LPS) markers, and elevated the expression of M(IL-4) markers. These data show a significantly altered lncRNA and mRNA expression profile in macrophages exposure to different activating conditions. Dysregulation of some of these lncRNAs may play important roles in regulating macrophage polarization.

Macrophages are an essential component of innate immunity and play a central role in inflammation and host defense^1^. In response to tissue microenvironmental signals contributed by microbial components, the innate and adaptive immune systems, and damaged cells and tissues, macrophages become activated and acquire diverse phenotypes and functions. The two states of macrophage activation, “Classical” and “Alternative” occupy two extremes of a phenotypic continuum in which macrophages respond to secreted factors to evoke distinct functional responses^2^,^3^.

Classically polarized activated macrophages have long been known to be induced by IFN-γ/LPS, or GM-CSF. M(IFN-γ + LPS) macrophages are characterized by high IL-12 and IL-23 and low IL-10 expression, by the production of nitric oxide (NO) by expressing inducible NO synthase (iNOS) and proinflammatory cytokines such as IL-6 and TNF-α, and by upregulation of molecules associated with antigen presentation such as HLA-DR and costimulatory molecules. Thus, M(IFN-γ + LPS) macrophages generally promote Th1-type inflammatory responses, and strong microbicidal and tumoricidal activity^4^,^5^. In contrast, the alternative M(IL-4) form of macrophage activation is a generic name used for various forms of non-classically activated macrophages resulting from cell exposure to IL-4 or IL-13, immune complexes, IL-10, or glucocorticoid. M(IL-4) macrophages express low levels of inflammatory cytokines such as IL-12 and high levels of the antiinflammatory cytokine IL-10, chemokines such as CCL17, CCL18, mannose receptors, and scavenger receptors. M(IL-4) macrophages have efficient phagocytic activity, but they are usually ineffective at killing microbial pathogens and are associated with anti-inflammatory and Th2 type responses, wound healing, and resolution of inflammation^6^,^7^. Thus, macrophage polarization is an important component of many disease states, including infection^8^, insulin resistance^9^, atherosclerosis^10^, cancer^11^, and autoimmune disease^12^. The identification of molecular mechanisms underlying macrophage plasticity and polarization provides a basis for macrophage-centered diagnostic and therapeutic strategies. However, the regulatory mechanisms controlling the expression of the constellation of genes in macrophage responding to activating conditions are not fully defined.

In recent years, Non-coding RNAs (ncRNAs) have emerged recently as key regulatory molecules with diverse roles in fundamental biological processes^13^,^14^. Based on their length, ncRNAs are classified into short ncRNAs (<200 nucleotides) and long non-coding RNAs (>200 nucleotides). The microRNA (miRNA) family of short ncRNAs are best characterised and are known to induce mRNA degradation or block mRNA translation via the RNA interference pathway^15^. In contrast to miRNAs, long noncoding RNAs (lncRNAs) are much less known concerning their functions. Recent evidence indicates that lncRNAs play essential roles in many cellular and developmental processes, including cell proliferation, apoptosis, and differentiation, as well as organ morphogenesis^16^,^17^,^18^. Aberrant expression of lncRNAs is closely associated with progression of pathophysiologic conditions including diabetes^19^, cancer^20^, tissue fibrosis^21^, and cardiovascular disease^22^. As with miRNAs, there is emerging evidence that lncRNAs are important regulators of the immune response in monocytes/macrophages^23^. However, the role of lncRNAs in the regulation of macrophage polarization have yet to be explored. A recent study suggested that lnRNAs are partially responsible for the coordinated changes in gene expression occurring during macrophage polarization^24^. So, we hypothesized that lncRNAs play a role in macrophage polarization.

To address our hypotheses, we employed a lncRNA microarray-based profiling assay to document changes in the abundance of lncRNAs induced by the activation of primary monocyte-derived macrophages (MDMs) with two distinct polarizing conditions to span the spectrum of described activation patterns [M(IFN-γ + LPS) and M(IL-4)]. Our data revealed that a number of lncRNAs and mRNAs were consistently altered under distinct polarizing conditions. More importantly, we demonstrated that TCONS_00019715 may play a critical role in promoting macrophage polarization to the M(IFN-γ + LPS) phenotype. These data suggest that lncRNAs are modifiers of macrophages gene expression that contribute to regulating macrophage gene expression responses to polarizing environmental conditions.

## Materials and Methods

### Human subjects

Human study protocols were approved by the ethical committee of the First Affiliated Hospital of Nanchang University and conducted in accordance with the Declaration of Helsinki. All participants provided informed consent before commencement of the study.

### Cell purification and culture

The peripheral blood samples were collected from healthy donors. After sample collection, peripheral blood mononuclear cells (PBMCs) were freshly isolated by density gradient centrifugation on Ficoll-Paque (Sigma-Aldrich, St Louis, USA) as described elsewhere. To develop MDMs, the monocytes were purified using anti-CD14 magnetic beads (Miltenyi Biotec, USA) and the purified monocytes were cultured in RPMI 1640 medium contains 10% Human serum and 0.05% Glutamine (Sigma) for 7 days at 5% CO_2_ and 37 °C. The resulted MDM was examined by morphology observation and FCM assay followed by anti-CD68 staining.

THP-1 cells were cultured in RPMI 1640 medium containing 10% FBS and 1.75 μL/500 mL β-mercaptoethanol (Sigma) and differentiated into macrophages by treatment with 5 ng/mL phorbol-12-myristate-13-acetate (PMA) (Sigma) overnight.

### Macrophage-polarizing conditions

To induce the polarization of macrophages, MDMs or PMA-differentiated THP-1 cells (THP-1 macrophages) were treated with 20 ng/mL of IFN-γ (R&D systems, USA) and 100 ng/mL of LPS (sigma) to achieve M(IFN-γ + LPS) polarization, or with 20 ng/mL of IL-4 (PeproTech, USA) to achieve M(IL-4) macrophages^25^. The non-polarized MDMs or THP-1 macrophages were cultured and left untreated [M(−)]. After the 18 hours polarization, the supernatant was collected and adherent cells were harvested for further analysis.

### Cytokine Analysis

The supernatant in cell culture plates was collected. The cytokines, TNF-α, IL-6, IL-12 (p70), IL-10, CCL17, CCL18 and CCL22 in the supernatants were measured with ELISA according to the manufacturer’s instructions. All ELISA kits were purchased from R&D Systems, USA.

### Isolation of RNA

Total RNA was extracted using TRIzol reagent (Invitrogen, Carlsbad, CA, USA) according to the manufacturer’s protocol. The integrity of the RNA was assessed by electrophoresis on a denaturing agarose gel. A NanoDrop ND-1000 spectrophotometer was used for the accurate measurement of RNA concentration.

### Microarray and computational analysis

Arraystar Human LncRNA Microarray v3.0 is designed for the global profiling of human LncRNAs and protein-coding transcripts. The sample preparation and microarray hybridization were performed based on the manufacturer’s standard protocols. Briefly, 10 qualitied mRNA samples of each group were purified from total RNA after removal of rRNA using an mRNA-ONLY^TM^ Eukaryotic mRNA Isolation Kit (Epicentre Biotechnologies, USA). Each sample was amplified and transcribed into fluorescent cRNA along the entire length of the transcripts without 3’ bias utilizing a random priming method. The concentration and specific activity of the labelled cRNAs (pmol Cy3/μg cRNA) were measured by NanoDrop ND-1000, 1 μg of each labelled cRNA was fragmented by adding 5 μL 10× blocking agent and 1 μL of 25× fragmentation buffer, heated to 60 °C for 30 min, and diluted with 25 μL 2 × GE hybridisation buffer. Fifty microlitres of hybridisation solution was dispensed into the gasket slide and assembled to the Human LncRNA Array v3.0 slide (8 × 60 K, Arraystar). The slides were incubated for 17 h at 65 °C in an Agilent hybridisation oven then washed, fixed and scanned using the Agilent DNA Microarray Scanner (part number G2505C). Approximately 30,586 lncRNAs and 26,109 coding transcripts collected from the most authoritative databases, such as RefSeq (release 55), UCSC Human (GRCh37/hg19), GENCODE 13, and lncRNAdb (2.0) were detected using microarray. Agilent Feature Extraction software (version 11.0.1.1) was used to analyze acquired array images. Quantile normalization and subsequent data processing were performed using the GeneSpring GX v12.0 software package (Agilent Technologies). Protein-coding genes were searched for differentially expressed lncRNAs using the UCSC Genome Browser (http://genome.ucsc.edu/cgi-bin/hgGateway). Genes transcribed within 300 kb were considered to represent nearby coding genes^26^. The microarray work was performed by KangChen Bio-tech, Shanghai, China.

### Quantitative real-time reverse transcription polymerase chain reaction

Quantitative real-time polymerase chain reaction (RT-qPCR) was used to confirm the expression pattern of selected lncRNAs in additional samples and macrophage polarization molecules. Expression of genes were analyzed by RT-qPCR using SYBR green reagents (Life Technologies, Carlsbad, CA) on an ABI 7500 instrument using indicated gene primers (Supplementary Table S1). All RT-qPCR experiments were performed in triplicate. All experiments included no-template controls. RT-qPCR data was analyzed by the 2^(−ΔΔCt)^ method, normalized against internal controls (GAPDH)^27^. PCR product was examined by agarose gel electrophoresis using 2% (w/v) LE agarose (Seakem) stained with ethidium bromide.

### Small interfering RNA

Two small interfering RNAs (siRNAs) against TCONS_00019715 (si-19715) at different sites and one negative control (si-NC) with no definite target were employed and synthesized by GenePharma (Shanghai, China). THP-1 macrophages were seeded on six-well plates at a density of 3 × 10^5^/well overnight, and then transfected with siRNA or the negative control at a final concentration of 50 nM using Lipofectamine 2000 (Invitrogen, USA). The interfering efficiency was determined by RT-qPCR 48 hours after transfection, and the siRNAs with silencing efficacy of more than 70% were selected for further experiments. Sequences of siRNAs and negative control are provided in Supplementary Table S1.

### Transfection of THP-1-derived macrophages

THP-1-derived macrophages were treated with with 20 ng/mL of IL-4 to achieve M(IL-4) macrophages. Cells were grown on six-well plates to 75% confluence and transfected with siRNA oligonucleotides using Lipofectamine 2000 (Invitrogen) according to the manufacturer’s protocol, and siRNAs were used at 50 nM final concentration. 48 hours after transfection, the supernatant was collected and adherent cells were harvested for further analysis.

### Statistical analysis

Each experiment was repeated at least three times. Numerical data were shown as the mean ± standard error of the mean (SEM). One-way ANOVA followed by the Bonferroni test was performed for multiple group comparisons. The Student t test was used for comparison between two groups. *P* < 0.05 was considered statistically signifiant.

## Results

### Identification of polarized MDM activation phenotypes

**Figure 1:**
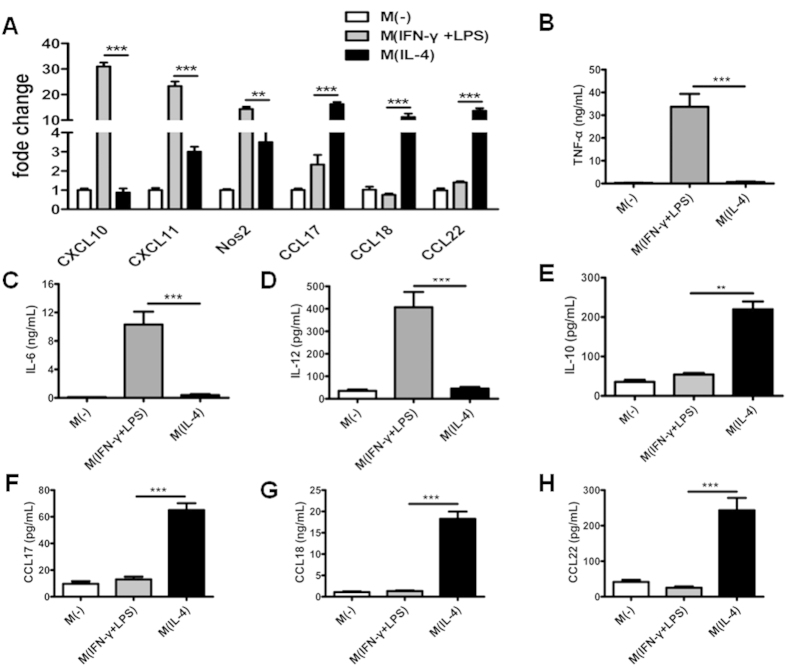
Identifiation of ex vivo-programmed M(IFN-γ + LPS) and M(IL-4) macrophages. MDMs were cultured in the presence of IFN-γ (20 ng/mL) plus LPS (100 ng/mL) or IL-4 (20 ng/mL). (**A**) Polarization-specific biomarkers were analyzed by RT-qPCR assays using RNA collected from MDMs at 18 hours post-treatment. (**B–H**) TNF-α (**B**), IL-6 (**C**), IL-12 (**D**), IL-10 (**E**), CCL17 (**F**), CCL18 (**G**) and CCL22 (**H**) in the supernatant were assayed by ELISA. Data are representative of three separate experiments, and show the means ± SEM. ^*^P < 0.05; ^**^P < 0.01; ^***^P < 0.001.

To screen for lncRNAs whose abundance was altered signifiantly following incubation of MDMs in two distinct polarizing conditions, we first generated M(IFN-γ + LPS) and M(IL-4) macrophages *in vitro*. We determined the phenotypic and functional markers associated with the human M(IFN-γ + LPS) and M(IL-4) macrophages, including Nos2, CXCL10, CXCL11, CCL17, CCL18, and CCL22 as well as the expressions of related cytokines in the culture supernatant, including TNF-α, IL-6, IL-12, IL-10, CCL17, CCL18 and CCL22. The results showed that with the IFN-γ/LPS induction, the expressions of M(IFN-γ + LPS) markers (Nos2, CXCL10 and CXCL11) were significantly increased, and the resulting proinflmmatory cytokine TNF-α, IL-6 and IL-12 were also significantly enhanced (Fig. 1). On the other hand, IL-4 induction in MDM up-regulated the expression levels of M(IL-4) molecular markers, such as CCL17, CCL18 and CCL22, and showed increased IL-10, CCL17, CCL18 and CCL22 production (Fig. 1). These data confimed that the polarization conditions used in this study resulted in distinct macrophage phenotypes.

### Expression profiles of lncRNAs in polarized MDMs

**Figure 2:**
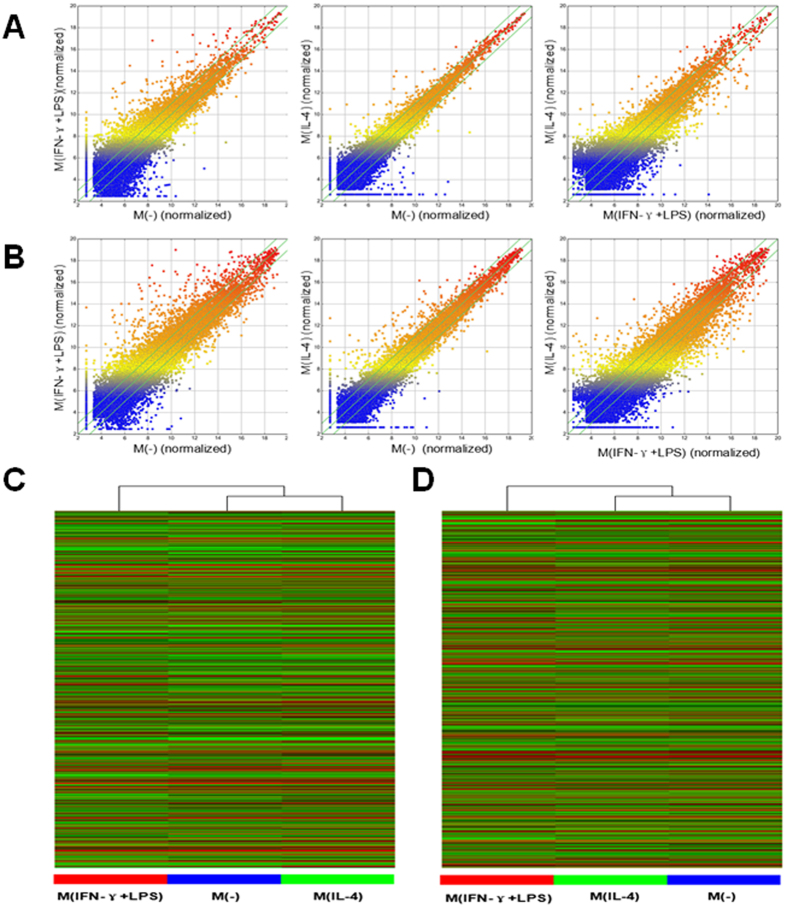
LncRNA and mRNA microarray expression data from MDMs incubated in distinct polarizing conditions. Scatter plots showing the variation in lncRNAs (**A**) and mRNAs (**B**) expression between the M(−), M(IFN-γ + LPS) and M(IL-4) macrophages. The values of the X and Y axes in the scatter plot are averaged normalized values in each group (log2-scaled). Hierarchical clustering results of lncRNAs (**C**) and mRNAs (**D**) expression profiles among three groups. “Red” indicated high relative expression and “green” indicated low relative expression. One ANOVA test was used for statistical analysis. LncRNA or mRNA with expression fold change >2 and with FDR adjusted *P* value <0.05 was considered statistically significant.

To study the potential biological functions of lncRNAs in regulating macrophage polarization, we examined the lncRNA and mRNA expression profiles in polarized MDMs through microarray analysis. For this analysis, authoritative data sources containing more than 30,586 lncRNAs were used. To identify the most significant candidates, lncRNAs with at least two fold expression change were selected (Fig. 2 and Supplementary Table S2). Under the criteria, 5820 lncRNAs were up-regulated and 3523 lncRNAs were down-regulated in the M(IFN-γ + LPS) group compared with the primary macrophages [M(−)] group. 2621 lncRNAs were increased and 1971 lncRNAs were decreased in the M(IL-4) group compared with the M(−) group. 4673 lncRNAs were overexpressed and 5807 lncRNAs were downexpressed in the M(IL-4) group compared with the M(IFN-γ + LPS) group. It was worth noting that there were significant difference in the expression levels of 326 lncRNAs between three groups. The majority of differentially expressed lncRNAs are from intergenic regions (~50%), natural antisense to protein-coding loci (~15%), or intronic antisense to protein-coding loci (~20%), with the others representing overlapping transcripts from exons or introns in both sense and antisense directions, exon sense-overlapping, or bidirectional regions.

### LncRNA classification and subgroup analysis

According to previous reports^14^,^16^, lncRNAs can be classified into different subgroups, such as lncRNAs with enhancer-like function (lncRNA-α), antisense lncRNA and large intergenic noncoding RNAs (lincRNAs). The expression profiles of 2252 intergenic lncRNAs indicated that they were differentially expressed (fold change ≥2.0, *P* < 0.05) between M(IL-4) group and M(IFN-γ + LPS) group (Supplementary Table S3). Among these, 1135 were upregulated and 1117 were downregulated. We also identified some nearby coding genes that may be regulated by these lncRNAs. LncRNAs with enhancer-like functions were identified using GENCODE annotation. The expression profiles of 1146 enhancer-like lncRNAs indicated that they were differentially expressed (fold change ≥2.0, *P* < 0.05) between M(IL-4) group and M(IFN-γ + LPS) group (Supplementary Table S4). Among these, 531 were upregulated and 615 were downregulated. We also identified some nearby coding genes that may be regulated by these enhancer-like lncRNAs. The expression profiles of 537 antisense lncRNAs indicated that they were differentially expressed (fold change ≥2.0, *P* < 0.05) between M(IL-4) group and M(IFN-γ + LPS) group (Supplementary Table S5). Among these, 237 were upregulated and 300 were downregulated. The number of lncRNAs differentially expressed in the subgroups [M(IFN-γ + LPS) vs. M(−), M(IL-4) vs. M(−), M(IL-4) vs. M(IFN-γ + LPS)] are listed in Table 1.

### Expression profiles of mRNAs in polarized MDMs

From the analysis, 3387 mRNAs were up-regulated and 2516 mRNAs were down-regulated in the M(IFN-γ + LPS) group compared with the M(−) group. 1121 mRNAs were increased and 2001 mRNAs were decreased in the M(IL-4) group compared with the M(−) group. 2528 mRNAs were overexpressed and 4534 mRNAs were downexpressed in the M(IL-4) group compared with the M(IFN-γ + LPS) group. It was worth noting that there were significant difference in the expression levels of 275 mRNAs between three groups. (Supplementary Table S6).

### Real-time quantitative PCR Validation

**Figure 3:**
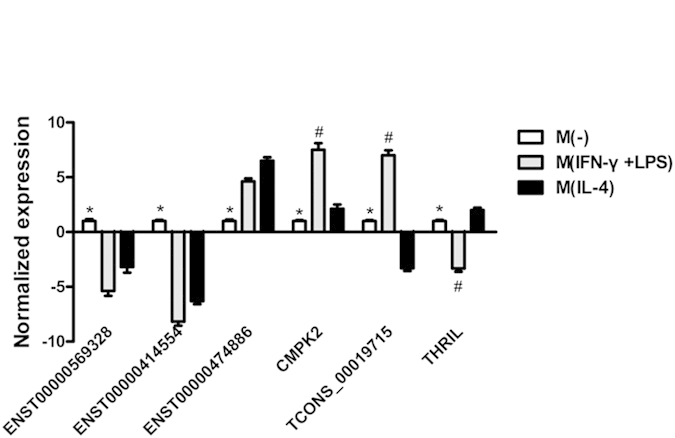
Confirmation of lncRNAs expression by RT-qPCR. Individual RT-qPCR assays were performed using samples from six additional MDM treated with polarizing conditions for 18 hours. After normalization to GAPDH expression, data were presented as mean ± SEM and obtained average expression value for each lncRNA was used for statistics. One ANOVA test for three groups or student’s t test for two groups was used for statistical analysis. Six lncRNAs were differentially expressed between three groups. ^*^Significant difference between M(−) group and M(IFN-γ + LPS) group, as well as between M(−) group and M(IL-4) group. ^#^Significant difference between M(IFN-γ + LPS) group and M(IL-4) group. *P* value <0.05 was considered statistically significant. Error bars in graphs referred to standard deviation. Each reaction was run three separate times, with technical triplicates in each reaction.

To confirm the microarray results, six lncRNAs were selected for further confirmation using RT-qPCR. A selection of these differentially expressed lncRNA transcripts that were selected for further studies are shown in Table 2. Replicate assays using MDMs from eight independent blood donors, showed that the abundance of ENST00000474886, or lncRNA- cytidine monophosphate kinase 2 (CMPK2) was significantly increased in MDMs exposed to M(IFN-γ + LPS), or M(IL-4) polarizing conditions, respectively(*P* < 0.05) (Fig. 3). Among the six selected lncRNAs, CMPK2, TCONS_00019715 and TNF-alpha and hnRNPL -related immunoregulatory lincRNA (THRIL) were differentially expressed between the M(IFN-γ + LPS) group and the M(IL-4) group (*P* < 0.05). The results were generally consistent with the microarray data.

Anticipating that it may be technically simpler to manipulate lncRNA function in a monocytic cell line than in primary macrophages (MDMs), we analyzed biomarkers and candidates lncRNAs in THP-1 macrophages (Supplementary Figure S1). Similar to MDMs, Nos2, CXCL10 and CXCL11 transcripts were predominantly induced in M(IFN-γ + LPS)-polarizing conditions, respectively, and induced to a lower level in the M(IL-4) -polarizing condition. CCL17, CCL18 and CCL22 transcripts were significantly increased in M(IL-4)-polarizing conditions. As such, we used this cell line as a model to test the function of lncRNAs up-regulated in M(IFN-γ + LPS) or M(IL-4) polarizing conditions. Six selected lncRNAs (ENST00000569328, ENST00000414554, ENST00000474886, CMPK2, TCONS_00019715 and THRIL) were regulated similarly in THP-1 macrophages and in MDMs exposed to M(IFN-γ + LPS) or M(IL-4) conditions (Supplementary Figure S2).

### Elevated expression of TCONS_00019715 in M(IFN-γ + LPS) polarized macrophages

**Figure 4:**
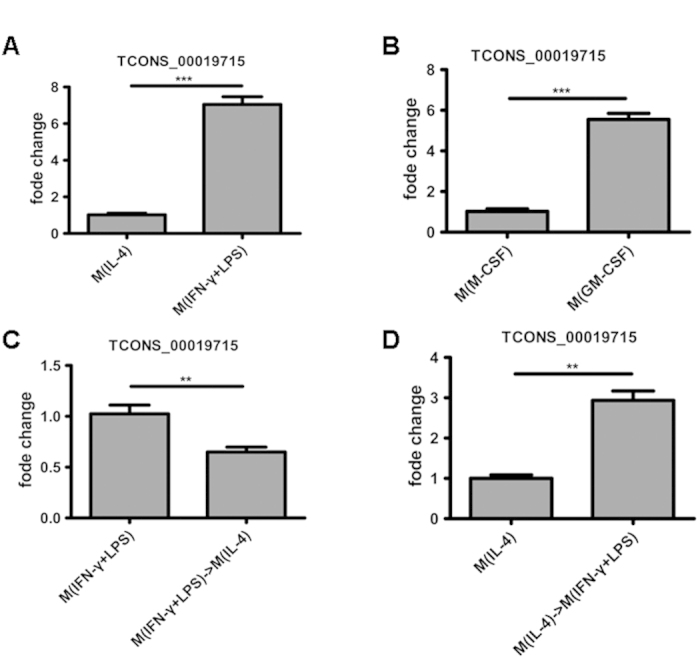
Differential expression of TCONS_00019715 during macrophage polarization. TCONS_00019715 was assessed by RT-qPCR and normalized to GAPDH in THP-1 macrophages after 18 hours of stimulation with IFN-γ (20 ng/mL) plus LPS (100 ng/mL) (**A**) or GM-CSF (20 ng/mL) (**B**). (**C**) TCONS_00019715 levels in macrophages following M(IFN-γ + LPS)-to-M(IL-4) re-polarization by IL-4 (20 ng/mL) for 18 hours. (**D**) TCONS_00019715 levels in macrophages following M(IL-4)-to-M(IFN-γ + LPS) re-polarization by IFN-γ (20 ng/mL) plus LPS (100 ng/mL) for 18 hours.

Among the differentially expressed lncRNAs among M(IFN-γ + LPS)-polarized macrophages and M(IL-4)-polarized macrophages, we were particularly interested in TCONS_00019715 because it had reduced expression under M(IL-4)-polarizing conditions, yet it had increased expression in M(IFN-γ + LPS) macrophages. The M(IFN-γ + LPS) macrophages have long been known to be induced by IFN-γ/LPS or GM-CSF. To account for possible differences in protocols for the *in vitro* differentiation of macrophages, we analyzed the amount of TCONS_00019715 in MDMs treated with GM-CSF and found that these were similar to those in IFN-γ/LPS-treated cells (Fig. 4). Therefore, we selected TCONS_00019715 for further characterization of its role in macrophage activation.

We next assessed the level of TCONS_00019715 in macrophages after the dynamic process of macrophage re-polarization. Macrophages with the M(IFN-γ + LPS) or M(IL-4) phenotypes were re-polarized to the M(IL-4) or M(IFN-γ + LPS) phenotype by treatment with IFN-γ/LPS or IL-4, respectively. The marker gene assays clearly showed that M(IFN-γ + LPS) macrophages could be re-polarized to M(IL-4) macrophages by IL-4 (Supplementary Figure S3A,B), whereas M(IL-4) macrophages could be re-polarized to M(IFN-γ + LPS) macrophages by IFN-γ/LPS (Supplementary Figure S3C,D). Interestingly, the TCONS_00019715 levels in macrophages were strikingly decreased following macrophages M(IFN-γ + LPS)-to-M(IL-4) re-polarization (Fig. 4C) but elevated during macrophages M(IL-4)-to-M(IFN-γ + LPS) re-polarization (Fig. 4D). The results suggest that TCONS_00019715 may play a critical role in promoting macrophage polarization to the M(IFN-γ + LPS) phenotype.

### Knockdown of TCONS_00019715 diminishes the expression of M(IFN-γ + LPS) phenotypes in M(IFN-γ + LPS) macrophages and promotes M(IFN-γ + LPS) macrophages transition to the M(IL-4) phenotype

**Figure 5:**
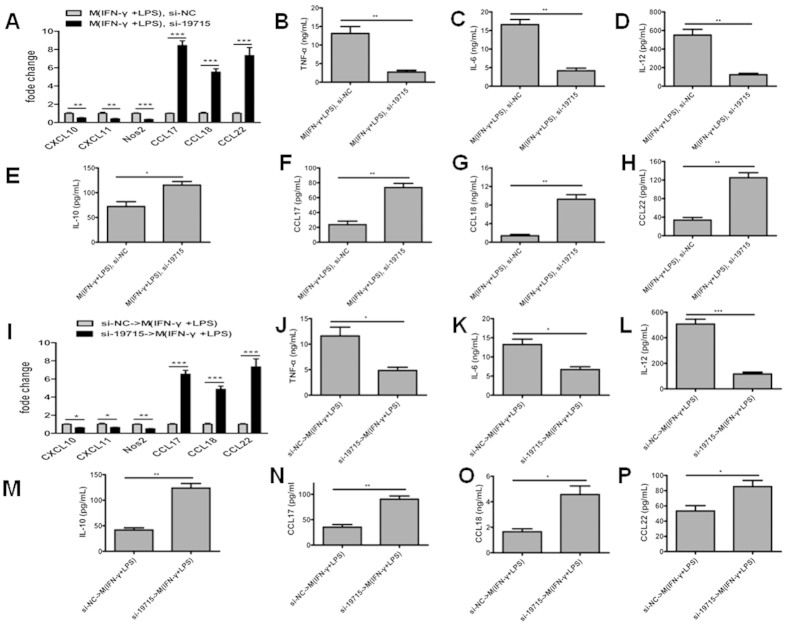
Knockdown of TCONS_00019715 promotes transition of M(IFN-γ + LPS) macrophages to the M(IL-4) phenotype and diminishes the expression of M(IFN-γ + LPS) phenotypes in M(IFN-γ + LPS) macrophages. Re-polarization of M(IFN-γ + LPS) macrophages to M(IL-4) macrophages by depleting TCONS_00019715 (**A**), correlating with reduction of TNF-α (**B**), IL-6 (C), IL-12 (**D**) and elevation of IL-10 (**E**), CCL17 (**F**), CCL18 (**G**) and CCL22 (**H**). Macrophages were transfected with an si-TCONS_00019715 (si-19715) or a control oligonucleotide (si-NC) and then stimulated with LPS and IFN-γ for M(IFN-γ + LPS) polarization (**I**), correlating with reduction of TNF-α (**J**), IL-6 (**K**), IL-12 (**L**) and elevation of IL-10 (**M**), CCL17 (**N**), CCL18 (**O**) and CCL22 (**P**). Data are representative of three separate experiments, and show the means ± SEM. ^*^P < 0.05; ^**^P < 0.01; ^***^P < 0.001.

To determine if TCONS_00019715 participates in macrophage polarization, we transfected M(IFN-γ + LPS) macrophages, which have high levels of TCONS_00019715 than do M(IL-4) macrophages, with siRNA for TCONS_00019715. Interestingly, we successfully re-polarized M(IFN-γ + LPS) macrophages from the M(IFN-γ + LPS) phenotype to the M(IL-4) phenotype, in which the M(IFN-γ + LPS) marker CXCL10, CXCL11 and Nos2 were down-regulated and the M(IL-4) markers CCL17, CCL18 and CCL22 were up-regulated (Fig. 5A–H). In addition, when primary macrophages were transfected with a siRNA-TCONS_00019715 and then treated with IFN-γ/ + LPS, the M(IFN-γ + LPS) polarization induced by IFN-γ and LPS was inhibited (Fig. 5I–P), suggesting that the promotion of macrophages M(IFN-γ + LPS) polarization by IFN-γ and LPS might act through the up-regulation of the TCONS_00019715 level.

## Discussion

In the present experiments we demonstrate that there is a significantly altered lncRNA and mRNA expression profile in distinct polarizing conditions. We have identified 326 lncRNA, and 275 protein coding transcripts with differential expression between M(−), M(IFN-γ + LPS) and M(IL-4) macrophages and we confirmed a selection of these differentially expressed transcripts by RT-qPCR. Furthermore, we found that TCONS_00019715 may play a critical role in promoting macrophage polarization to the M(IFN-γ + LPS) phenotype. To our knowledge, this is the first report on the expression of lncRNAs in human macrophages with polarized phenotypes.

Macrophages are dynamic and heterogeneous cells whose phenotypes and functions are shaped by microenvironmental signals. M(IFN-γ + LPS) and M(IL-4) cells represent extremes of a continuum of macrophages heterogeneity^4^. Tremendous progress has been made in defining the molecular networks underlying M(IFN-γ + LPS)-M(IL-4)-polarized activation of macrophages^28^. However, new molecules that regulate macrophages M(IFN-γ + LPS)-M(IL-4) polarization may still remain unidentified. LncRNAs play an important role in many biological processes, including X-chromosome inactivation, gene imprinting, and stem cell maintenance. Expression profiling experiments have documented changes in lncRNAs expression in human and murine monocytic cells responding to selected inflammatory conditions^29^,^30^,^31^. In these experiments, a subset of lncRNAs has been repeatedly documented to be induced following inflammation-inducing stimuli, which induce differentiation toward the inflammation phenotype^32^. We reasoned that lncRNAs may be involved not only in macrophages responses to inflammatory conditions but also in the modifications of gene expression required to generate a spectrum of macrophage activation patterns. To better represent the spectrum, therefore, our study was designed to identify lncRNAs that respond to stimuli inducing two major patterns of macrophage activation [M(IFN-γ + LPS) and M(IL-4)].

In this study, we analyzed lncRNA expression profiles in the polarized MDMs to uncover the potential role of lncRNAs in the macrophage polarization. Highthroughput microarray techniques revealed a set of differentially expressed lncRNAs, including 4673 that were upregulated and 5807 that were downregulated in M(IL-4) macrophages compared to M(IFN-γ + LPS) macrophages. LncRNAs are usually divided into five categories: sense, antisense, bidirectional, intronic, and intergenic. Here, we found that most deregulated lncRNAs were from intergenic regions (~50%), natural antisense to protein-coding loci (~15%), or intronic antisense to protein-coding loci (~20%). Therefore, the data shown here provides a comprehensive profile of the lncRNAs and coding transcript expression in the macrophages with polarized phenotypes.

Subsequently, Six lncRNAs (CMPK2, THRIL, TCONS_00019715, ENST00000569328, ENST00000414554 and ENST00000474886) were selected from the differentially expressed lncRNAs for further analysis in this study. CMPK2, THRIL were selected because of their roles in modulating inflammatory response of macrophages^30^,^33^. TCONS_00019715 was selected because it locates near PAK1 gene, which codes a protein that was shown to modulate macrophage polarization. The other 3 lncRNAs were randomly selected. Data showed that RT-qPCR results were generally consistent with the microarray data. Recently, Kambara *et al*.^33^ found that lncRNA-CMPK2 is regulated by the JAK-STAT signaling pathway, and that it is involved in the downregulation of the interferon response. We found that M(IFN-γ + LPS) macrophages exhibit a considerably higher level of lncRNA-CMPK2 than no treatment macrophages and M(IL-4) macrophages. LncRNA THRIL is located downstream of the gene encoding the BRI3 binding protein (Bri3bp), is transcribed from the opposite strand, and partially overlaps the 3’-end of Bri3bp. Li *et al*.^30^ found that Pam3CSK4 stimulation decreased the expression of THRIL and knockdown of THRIL notably restrained TNF-α secretion. As with lincRNA-Cox2^31^, THRIL was shown to interact with hnRNPL, with the resultant complex binding to the TNF-α promoter and driving transcription in both control and Pam3CSK4-stimulated THP1 macrophages. Our results showed that THRIL was down-regulated in M(IFN-γ + LPS) macrophages compared with no treatment macrophages. These data suggested that lncRNA-CMPK2 and THRIL may be involved in macrophages responses to inflammatory conditions.

Of these six lncRNAs, only TCONS_00019715, which is a 286-bp transcript and is located in the chr11. on the forward strand, was strongly induced expression under M(IFN-γ + LPS)-polarizing conditions, yet it had decreased expression in M(IL-4) polarized macrophages. To examine whether TCONS_00019715 contributes to the plasticity of macrophage polarization, we attempted to convert one population into another by culturing M(IFN-γ + LPS) macrophages with IL-4 and M(IL-4) macrophages with IFN-γ/LPS. Our experiments demonstrated that M(IFN-γ + LPS)-to-M(IL-4) conversion resulted in decreased TCONS_00019715, whereas M(IL-4)-to-M(IFN-γ + LPS) led to increased TCONS_00019715 expression. It was surprising that knockdown of TCONS_00019715 diminishes the expression of M(IFN-γ + LPS) phenotypes in M(IFN-γ + LPS) macrophages and promotes M(IFN-γ + LPS) macrophages transition to the M(IL-4) phenotype. These data suggest that TCONS_00019715 may play an important role in promoting macrophage polarization to the M(IFN-γ + LPS) phenotype.

LncRNAs are known to function via a variety of mechanisms; however, a common and important function of lncRNAs is to alter the expression of nearby encoding genes by affecting the process of transcription^34^,^35^. Several reports have demonstrated that the expression levels of certain lncRNAs are correlated with the nearest protein-coding genes and that these lncRNAs may have either a positive^36^ or negative^37^ effect on gene expression. Accordingly, we were interested in the genes surrounding TCONS_00019715. We then retrieved the genomic locus information and found that the protein-coding gene PAK1 is close to it. Serine/threonine kinase family members p21-activated kinases (PAKs) are important regulators of cytoskeletal remodeling and cell motility in mononuclear phagocytic system. Recently, Zhang *et al*.^38^ found that inflammatory stimuli induced PAK1 overexpression in human and murine macrophages and elevated expression of PAK1 contributed to macrophages M(IFN-γ + LPS) polarization. In our study, we found that the PAK1 mRNA levels were increased in M(IFN-γ + LPS) MDMs (Supplementary Figure S4A,B). Treatment of macrophages with IFN-γ + LPS or GM-CSF resulted in upregulation of PAK1 mRNA expression in MDMs and THP-1 macrophages. It was reported that knockdown or down-regulation of certain lncRNAs could lead to decreased expression of its neighboring protein-coding gene, which suggested that lncRNAs and nearby coding genes could have shared upstream regulation or local transcriptional effects^14^. Accordingly, we have found that TCONS_00019715 knockdown could downregulate PAK1 mRNA expression (Supplementary Figure S4C,D). Moreover, Knockdown of PAK1 in M(IFN-γ + LPS) macrophages diminished the expression of M(IFN-γ + LPS) markers, and elevated the expression of M(IL-4) markers (Supplementary Figure S5). These data demonstrated that PAK1 may be involved in mediating the effects of TCONS_00019715 on macrophage polarization. Future studies using ChIRP (chromatin isolation by RNA purification) assays, knockdown or over-expression techniques in a relevant model system would be a reasonable approach to uncover their potential regulatory mechanisms in macrophage polarization.

In summary, this study has examined the global expression patterns of lncRNAs in macrophage polarization and contributed to the growing understanding of the role of lncRNAs in macrophages exposure to different activating conditions. Thus, lncRNA profiing reveals novel molecules and signatures associated with differentiation of macrophages and polarized activation which may be candidate targets in pathophysiology. Moreover, we found that TCONS_00019715 may plays an important role in regulating macrophage polarization. Further investigation of the lncRNAs identified in this study will likely shed light on their biological functions and their association with macrophage polarization.

## Additional Information

**How to cite this article**: Huang, Z. *et al*. Identification of Differentially Expressed Long Non-coding RNAs in Polarized Macrophages. *Sci. Rep*. **6**, 19705; doi: 10.1038/srep19705 (2016).

## Supplementary Material

Supplementary Information

Supplementary Table S2

Supplementary Table S3

Supplementary Table S4

Supplementary Table S5

Supplementary Table S6

## Figures and Tables

**Table 1:** The number of lncRNAs differentially expressed in the subgroups (fold change ≥2.0).

Comparison		Up-regulation	Down-regulation
M(IFN-γ + LPS) vs. M(−)	lincRNAs	1075	607
	enhancer-like lncRNAs	525	340
	antisense lncRNAs	303	155
M(IL-4) vs. M(−)	lincRNAs	343	132
	enhancer-like lncRNAs	152	98
	antisense lncRNAs	78	55
M(IL-4) vs. M(IFN-γ + LPS)	lincRNAs	1135	1117
	enhancer-like lncRNAs	531	615
	antisense lncRNAs	237	300

**Table 2:** LncRNAs differentially expressed in polarized macrophages determined by microarray (Arraystar Human LncRNA Microarray v3.0) selected for validation studies.

Seqname	Fold change (absolute)			GeneSymbol	RNAlength	Chromsome	Strand	Relationship
	M(IFN-γ + LPS) vs. M(−)	M(IL-4) vs. M(−)	M(IL-4) vs. M(IFN-γ + LPS)					
ENST00000569328	−6.46	−3.85	ns	RP11-700H13.1	445	chr16	−	intergenic
ENST00000414554	−8.48	−7.58	ns	AC018737.1	557	chr2	+	bidirectional
ENST00000474886	2.76	3.65	ns	CFLAR-AS1	223	chr2	−	natural antisense
CMPK2	6.31	2.31	−4.89	AC017076.5	506	chr2	−	intergenic
TCONS_00019715	6.35	−2.11	−8.69	XLOC_009510	286	chr11	−	exon sense-overlapping
THRIL	−2.95	ns	2.36	XLOC_010236	2893	chr12	−	natural antisense

CMPK2: cytidine monophosphate kinase 2 (CMPK2).

THRIL: TNF-alpha and hnRNPL -related immunoregulatory lincRNA.
