# Supplementary material for: Identification of Differentially Expressed Long Non-coding RNAs in Polarized Macrophages
Source: Sci Rep. 2016 Jan 22;6:19705. doi: 10.1038/srep19705 (PMC4726337; doi:10.1038/srep19705)
Supplement: Supplementary Information [file srep19705-s1.pdf]

Identification of Differentially Expressed Long Non-coding RNAs in Polarized Macrophages

Zikun Huang, Qing Luo, Fangyi Yao, Cheng Qing, Jianqing Ye, Yating Deng, Junming Li*

Supplementary Table 1. RT-qPCR primers and siRNA sequences

Name		Sequences
qPCR primers		
GAPDH	Sense	5'-GCACCGTCAAGGCTGAGAAC-3'
	Anti-sense	5'-TGGTGAAGACGCCAGTGGA-3'
CXCL10	Sense	5'-GGCCATCAAGAATTTACTGAAAGCA-3'
	Anti-sense	5'-TCTGTGTGGTCCATCCTTGGAA-3'
CXCL11	Sense	5'-CCTTGGCTGTGATATTGTGTGCTA-3'
	Anti-sense	5'-CCTATGCAAAGACAGCGTCCTC-3'
CCL17	Sense	5'-TGAGGACTGCTCCAGGGATG-3'
	Anti-sense	5'-AACGGTGGAGGTCCCAGGTA-3'
CCL18	Sense	5'-AAGAGCTCTGCTGCCTCGTCTA-3'
	Anti-sense	5'-CCCTCAGGCATTCAGCTTCA-3'
NOS2	Sense	5'-GCCAAGCTGAAATTGAATGAGGA-3'
	Anti-sense	5'-TTCTGTGCCGGCAGCTTTAAC-3'
TCONS_00019715	Sense	5'-AGATGCGGTGGTGCTTTG-3'
	Anti-sense	5'-GGACGATTTCTGGGAGGG-3'
ENST00000569328	Sense	5'-GGCAGCCGAACAACAGAA-3'
	Anti-sense	5'-TGGCAGGGAAAGCAAACA-3'
ENST00000414554	Sense	5'-CGGAAAGTGTTTTGGAGC-3'
	Anti-sense	5'-AATTACCGGCGTGCATAG-3'
ENST00000474886	Sense	5'-CCCTGAAGTTATTTGAAGGATCC-3'
	Anti-sense	5'-CTCAGTACCTCCCTTCTGCTTTT-3'
CMPK2	Sense	5'-CCAGGTTGTTGCCATCGAAG-3'
	Anti-sense	5'-CAAGAGGGTGGTGACTTTAAGAG-3'
THRIL	Sense	5'-GGTCTCAATCCACCAGCACCCAG-3'

	Anti-sense	5'-TCCTCCCACCCTCTTACCCGAAA-3'
PAK1	Sense	5'-TATGATTGGAGCCGGCAGC-3'
	Anti-sense	5'-CTGAAGGGAGAGAAATCTCTGGC-3'
siRNA		
PAK1	siRNA1	5'-GAAAGAGCGGCCAGAGAUU-3'
	siRNA2	5'-GCAUCAAUUCCUGAAGAUU-3'
TCONS_00019715	siRNA	5'-GCUGUGAGAUCACACGACA-3'

Supplementary Figure Legends

Figure S1. Identifiation of ex vivo-programmed M(IFN-γ+LPS) and M(IL-4) macrophages. THP-1 macrophages were cultured in the presence of IFN-γ (20 ng/mL) plus LPS (100 ng/mL) or IL-4 (20 ng/mL). (A) Polarization-specific biomarkers were analyzed by RT-qPCR assays using RNA collected from MDMs at 18 hours post-treatment. (B) TNF-α, IL-6, IL-12, IL-10, CCL17, CCL18 and CCL22 in the supernatant were assayed by ELISA. Data are representative of three separate experiments, and show the means ± SEM. $^{*}P<0.05$; $^{**}P<0.01$; $^{***}P<0.001$.

Figure S2. Confirmation of lncRNAs expression by RT-qPCR. Individual RT-qPCR assays were performed using samples from THP-1 macrophages treated with polarizing conditions for 18 h. After normalization to GAPDH expression, data were presented as mean ± SEM and obtained average expression value for each lncRNA was used for statistics. One ANOVA test for three groups or student's t test for two groups was used for statistical analysis. Six lncRNAs were differentially expressed between three groups. *Significant difference between M0 group and M(IFN-γ+LPS) group, as well as between M0 group and M(IL-4) group. $^{\#}$Significant difference between M(IFN-γ+LPS) group and M(IL-4) group. Data are representative of three separate experiments, and show the means ± SEM. $^{*}P<0.05$; $^{**}P<0.01$; $^{***}P<0.001$.

Figure S3. Relative gene expression and protein level during macrophage

M(IFN-γ+LPS)/ M(IL-4) polarization. Gene expression (A) and levels of IL-10 or IL-12 (B) following re-polarization of M(IFN-γ+LPS) macrophages to M(IL-4) macrophages by LPS/IFN-γ. Gene expression (C) and levels of IL-4 (D) following re-polarization of M(IL-4) macrophages to M(IFN-γ+LPS) macrophages by LPS/IFN-γ. Data are representative of three separate experiments, and show the means ± SEM. $^{*}P<0.05$; $^{**}P<0.01$; $^{***}P<0.001$.

Figure S4. PAK1 expression is augmented by inflammatory stimuli in macrophages. PAK1 was assessed by RT-qPCR and normalized to GAPDH in THP-1 macrophages after 18 hours of stimulation with IFN-γ (20 ng/mL) plus LPS (100 ng/mL) (A) or GM-CSF (20 ng/mL) (B). PAK1 mRNA levels were assessed by RT-qPCR and normalized to GAPDH in MDMs (C), and THP-1 macrophages (D) after 48 hours of transfection with an si-TCONS_00019715 or a control oligonucleotide. Data are representative of three separate experiments, and show the means ± SEM. $^{*}P<0.05$; $^{**}P<0.01$; $^{***}P<0.001$.

Figure S5. Knockdown of PAK1 promotes transition of M(IFN-γ+LPS) macrophages to the M(IL-4) phenotype and diminishes the expression of M(IFN-γ+LPS) phenotypes in M(IFN-γ+LPS) macrophages. qRT-PCR revealed that PAK1 was efficiently knocked down by treatment with si-PAK1 in M(IFN-γ+LPS) macrophages (A). Re-polarization of M(IFN-γ+LPS) macrophages to M(IL-4) macrophages by depleting PAK1 (B), correlating with reduction of IL-12 and elevation of IL-10 (C), Macrophages were transfected with an si-PAK1 or a control oligonucleotide (si-NC) and then stimulated with LPS and IFN-γ for M(IFN-γ+LPS) polarization (D), correlating with elevation of IL-10 and reduction of IL-12 (E). Data are representative of three separate experiments, and show the means ± SEM. $^{*}P<0.05$; $^{**}P<0.01$; $^{***}P<0.001$.

Figure S1.

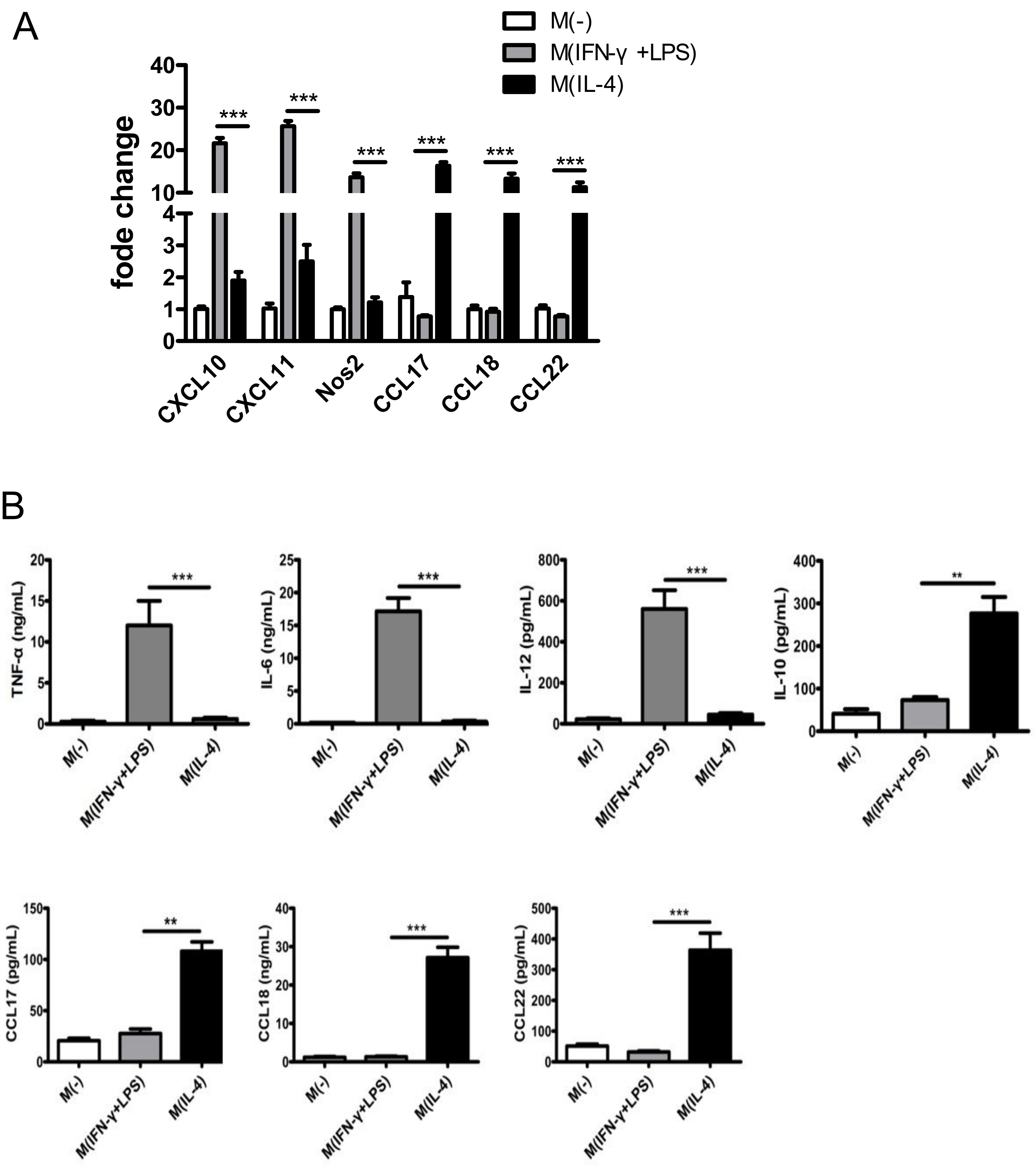
A
M(-)
M(IFN-γ +LPS)
M(IL-4)
fode change
40
30
20
10
4
3
2
1
0
***
CXCL10
CXCL11
Nos2
CCL17
CCL18
CCL22
B
TNF-α (ng/mL)
IL-6 (ng/mL)
IL-12 (pg/mL)
IL-10 (pg/mL)
CCL17 (pg/mL)
CCL18 (ng/mL)
CCL22 (pg/mL)
***
**
M(-)
M(IFN-γ+LPS)
M(IL-4)

Figure S2.

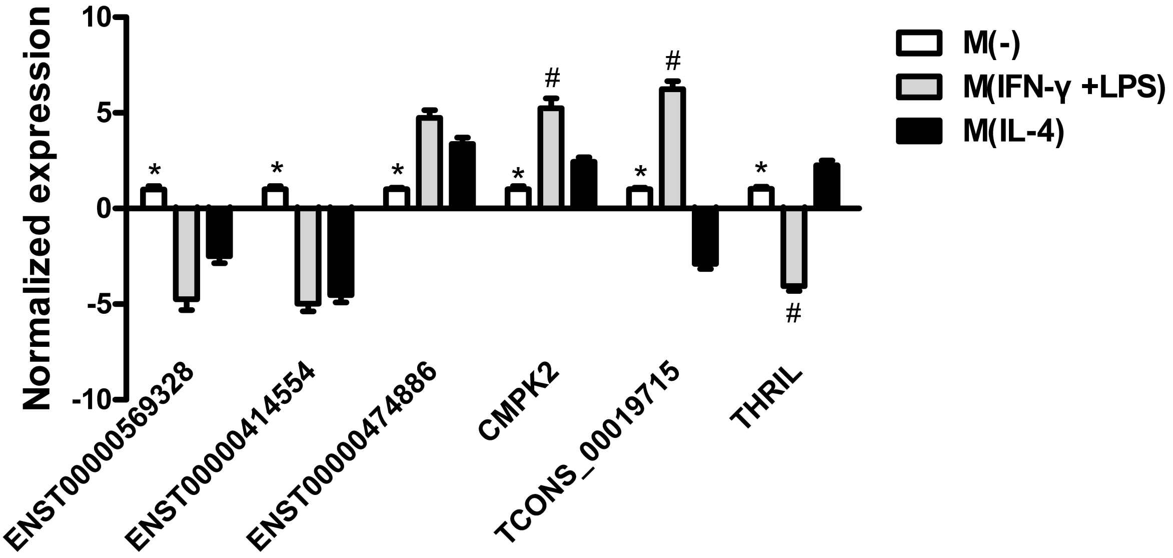
Normalized expression
10
5
0
-5
-10
M(-)
M(IFN-γ +LPS)
M(IL-4)
*
#
ENST00000569328
ENST00000414554
ENST00000474886
CMPK2
TCONS_00019715
THRIL

Figure S3.

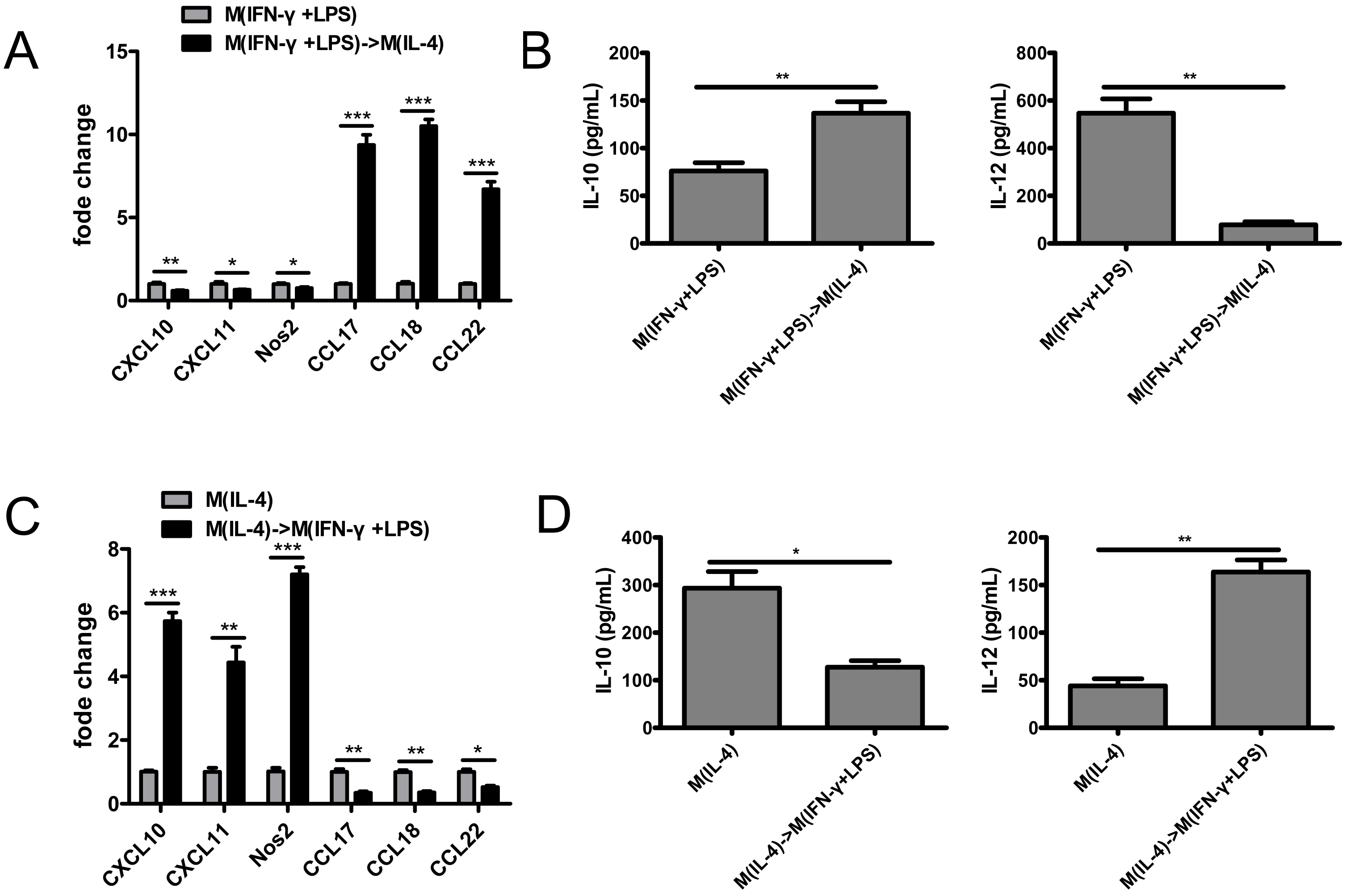
A
M(IFN-γ +LPS)
M(IFN-γ +LPS)->M(IL-4)
fode change
15
10
5
0
**
*
*
***
***
***
CXCL10
CXCL11
Nos2
CCL17
CCL18
CCL22
B
IL-10 (pg/mL)
200
150
100
50
0
**
M(IFN-γ+LPS)
M(IFN-γ+LPS)->M(IL-4)
IL-12 (pg/mL)
800
600
400
200
0
**
M(IFN-γ+LPS)
M(IFN-γ+LPS)->M(IL-4)
C
M(IL-4)
M(IL-4)->M(IFN-γ +LPS)
fode change
8
6
4
2
0
***
**
***
**
**
*
CXCL10
CXCL11
Nos2
CCL17
CCL18
CCL22
D
IL-10 (pg/mL)
400
300
200
100
0
*
M(IL-4)
M(IL-4)->M(IFN-γ+LPS)
IL-12 (pg/mL)
200
150
100
50
0
**
M(IL-4)
M(IL-4)->M(IFN-γ+LPS)

Figure S4.

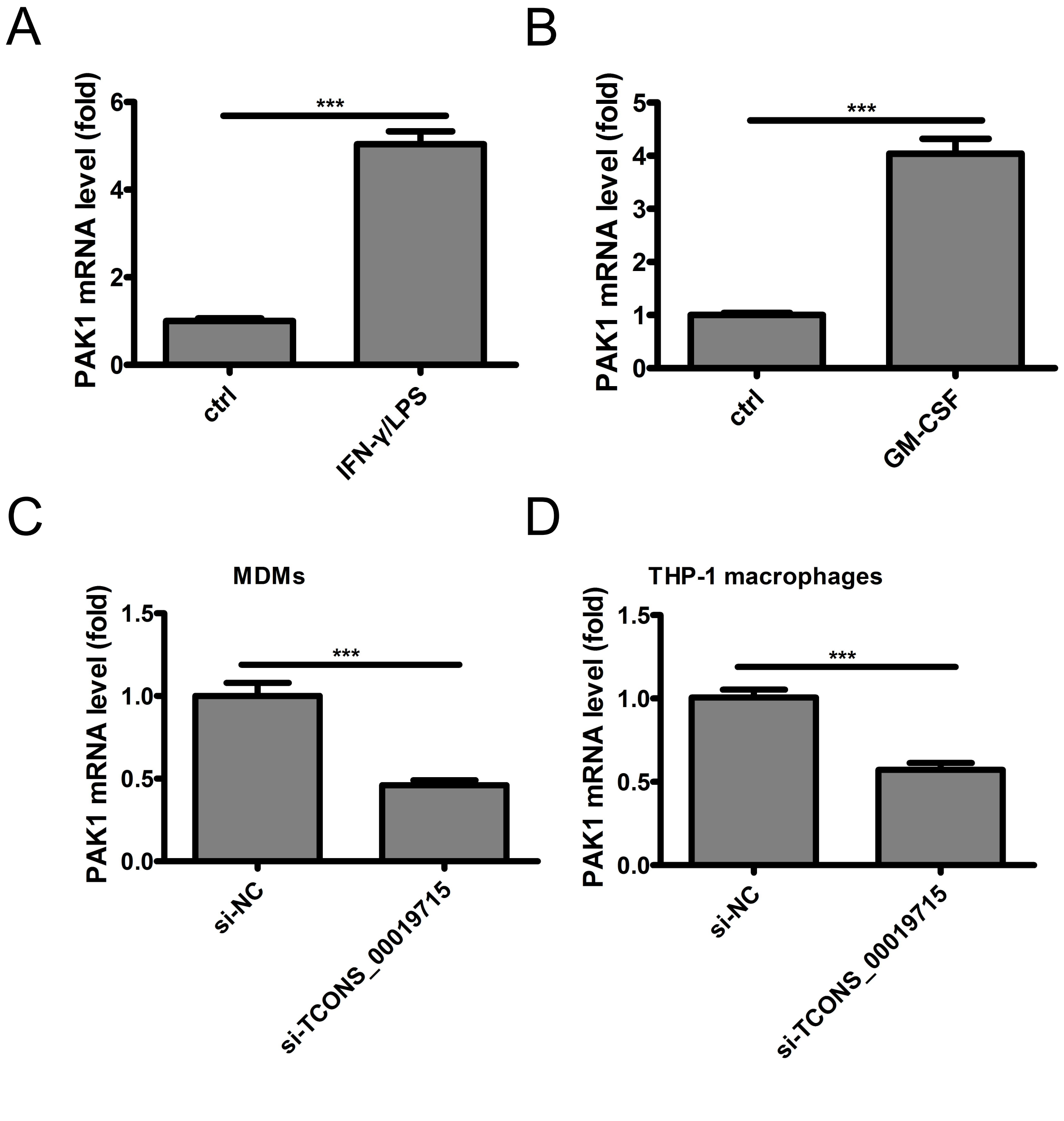
A
B
C
D
PAK1 mRNA level (fold)
***
ctrl
IFN-γ/LPS
GM-CSF
MDMs
THP-1 macrophages
si-NC
si-TCONS_00019715
0
2
4
6
1
3
5
0.0
0.5
1.0
1.5

Figure S5.

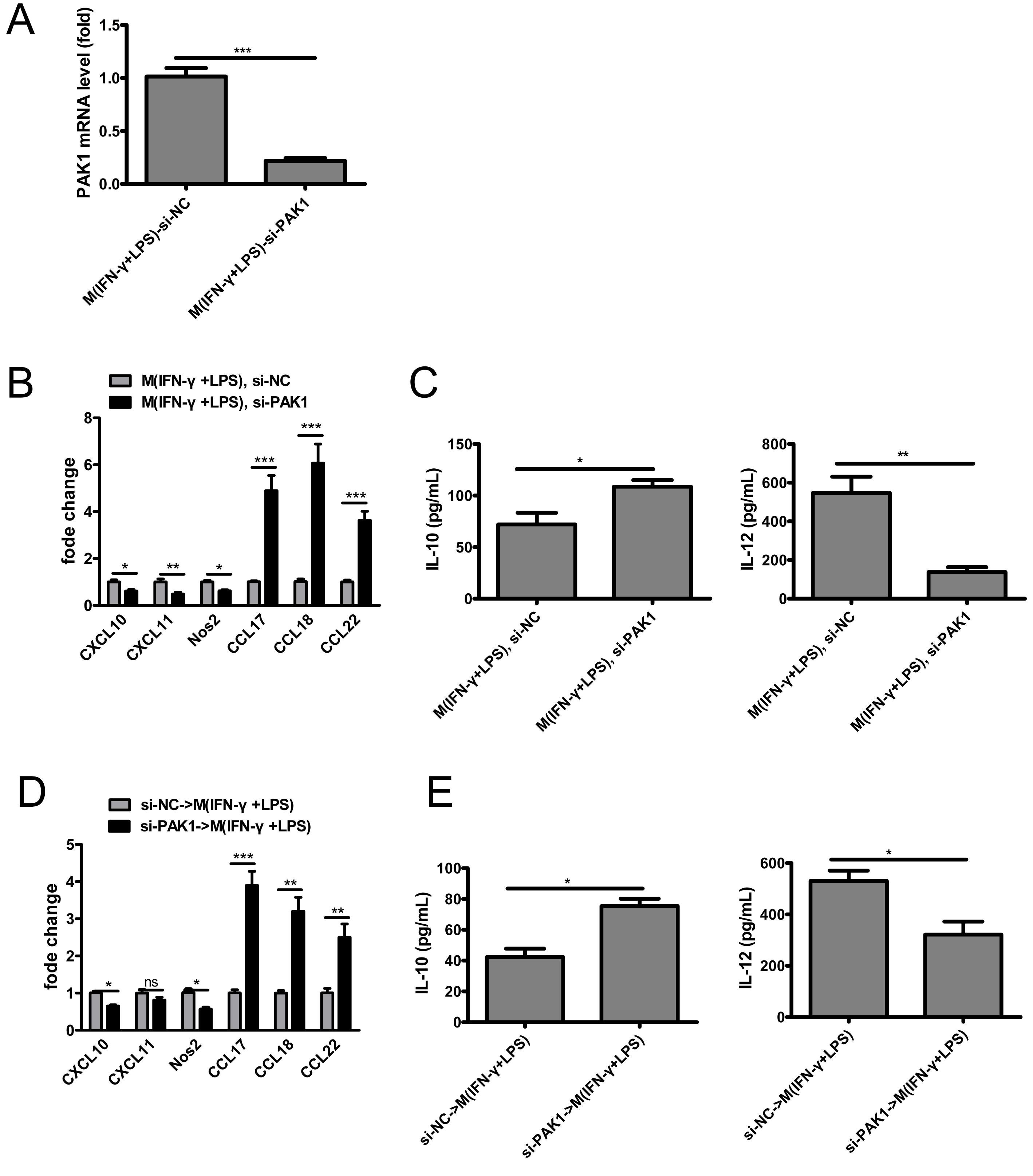
A
PAK1 mRNA level (fold)
1.5
1.0
0.5
0.0
***
M(IFN-γ+LPS)-si-NC
M(IFN-γ+LPS)-si-PAK1
B
M(IFN-γ +LPS), si-NC
M(IFN-γ +LPS), si-PAK1
fode change
CXCL10
CXCL11
Nos2
CCL17
CCL18
CCL22
C
IL-10 (pg/mL)
IL-12 (pg/mL)
M(IFN-γ+LPS), si-NC
M(IFN-γ+LPS), si-PAK1
D
si-NC->M(IFN-γ +LPS)
si-PAK1->M(IFN-γ +LPS)
fode change
ns
E
IL-10 (pg/mL)
IL-12 (pg/mL)
si-NC->M(IFN-γ+LPS)
si-PAK1->M(IFN-γ+LPS)
